# Supplementary material for: Vertical integration of primary care practices with acute hospitals in England and Wales: why, how and so what? Findings from a qualitative, rapid evaluation
Source: BMJ Open. 2022 Jan 11;12(1):e053222. doi: 10.1136/bmjopen-2021-053222 (PMC8753412; doi:10.1136/bmjopen-2021-053222)
Supplement: Supplementary data [file bmjopen-2021-053222supp001.pdf]

Supplementary File 1: Interview topic guide

1. Can you please describe your current role within your NHS organisation?
   - Length of time in their current role
   - Their key responsibilities
2. What was the rationale for your acute trust/health board taking over and directly managing general practice services in your area?
   - Who initiated this model of vertical integration?
   - What was the rationale/drivers from the perspective of the acute trust/health board?
   - What was the rationale/drivers from the perspective of general practices?
   - What concerns were raised? By whom?
   - What other models, if any, were considered e.g. horizontal integration?
   - Has the rationale changed since the model was first implemented?
3. Could you please describe the current model of the acute trust or health board taking over and directly managing general practice services in your area?
   - Length of time since first practice was directly managed
   - How are practices selected to join the model?
   - Current stakeholders involved
   - Number of practices and any increase or decrease since implementation
   - Against what criteria are decisions about which practices can join/be part of the integration made?
   - Why have some practices decided to leave or join?
4. How does this model of vertical integration impact upon governance, contractual and legal arrangements amongst general practices? And, between the acute trust/health board and the general practices?
   - Who's holding the AMS/GMS contract?
   - Is there any governance of this arrangement?
   - What changes have occurred (if any) with regard to indemnity?
   - Who now owns/leases general practice properties?
   - Creation of a limited company (Somerset)?
   - What about risk management both on behalf of the acute trust/health board and general practices themselves?
5. Could you please describe the process of implementing VI in your area?

- What were the first steps taken to initiate the implementation of VI? How has the process evolved?
- Who was involved in the implementation process (from the acute trust/health board, primary care/general practices, and the wider system)?
- Have there been issues of tension amongst stakeholders?
- Introduction of a new board over-seeing management/performance of integration – who is involved? Frequency of meetings?
- Sharing of 'back office' functions?

6. What have been the financial implications for the acute trust/health board and for the general practices they manage?
   - What are the financial incentives?
   - What have been the one-off cost implications/ on-going costs for trusts/boards?
   - The financial performance of general practices
   - Are there any financial drawbacks of this model of integration?
7. How is 'success' being determined and/or measured?
   - Process measures
   - Intended outcomes (clinical and non-clinical)
   - Unintended outcomes
   - How is progress and impact of this integration being tracked (using what data) for the short and long term?
8. What has changed at the level of health service provision?
   - How has the delivery of services changed in given settings (primary and secondary care, and wider community services)?
   - What has been the impact on patients accessing services across the care interface?
   - Has integration led to changes to how certain groups of patients are managed?
   - Does the impact differ for patients from VI practices compared to those registered with non-VI practices? Are patients experiencing something new/different?
   - What changes have there been with regard to referral pathways/processes? (if not now, will it change in the future?)
9. What are the risks involved with this model of VI?
   - Have there been any unintended consequences of introducing this model of vertical integration?
   - How does the introduction of VI align with the delivery of primary and secondary care services within your area?
     a. Integrated care systems

b. Primary care networks/ test bed sites

10. What has been the impact upon practice staff or staffing?
    - What has been the impact of the recruitment and retention across both primary and secondary care?
    - Introduction of new personnel: new types of staff/roles; additional staff numbers?
    - Has the introduction of VI impacted upon career progression? E.g. early career GPs
    - Has working within a VI model had any impact upon job satisfaction amongst staff?
    - What have been some of the key changes which staff have noticed in their everyday working practices?
11. To your knowledge, have any other trusts/health boards expressed interest in adopting a similar vertical integration model?
    - What advice would you give such prospective areas if they were thinking about adopting this model of VI?
    - Do you think VI is an attractive (or viable) model for the NHS in the UK and does it have potential for 'scale up'?
12. What are the main priorities for your acute trust/health board or practice for the future?
    - How are they planning to further develop the VI model (what are the next steps)?
13. Is there anything else you wanted to tell us about that has not already been covered in the interview?
