# Supplementary material for: Vertical integration of primary care practices with acute hospitals in England and Wales: why, how and so what? Findings from a qualitative, rapid evaluation
Source: BMJ Open. 2022 Jan 11;12(1):e053222. doi: 10.1136/bmjopen-2021-053222 (PMC8753412; doi:10.1136/bmjopen-2021-053222)
Supplement: Supplementary data [file bmjopen-2021-053222supp002.pdf]

Supplementary File 2: Coding framework

Integrative themes	Sub-codes	Description of sub-codes	Research question
Understanding the need and purpose of integration with primary care			RQ1
	Change in rationale	Has the rationale changed since the model was first implemented?	RQ1
	Concerns	What concerns were raised? By whom?	RQ1
	Initiation	Who initiated this model of vertical integration?	RQ1
	Other models	Were any other models considered? Which ones?	RQ1
	Rationale	What was the rationale from the perspective of the acute trust/health board and/or general practices?	RQ1
	Scale up	Do you think vertical integration is an attractive (or viable) model for the NHS in the UK and does it have potential for 'scale up'?	RQ1

Integrative themes	Sub-codes	Description of sub-codes	Research question
Developing an integration model and implementation strategy for general practices			RQ2
	Alignment of vertical integration 1	How does the introduction of vertical integration align with the delivery of primary and secondary care services within your area? - Integrated care systems - Primary care networks	RQ2
	PCN future	The future of the current vertical integration model in the context of developing PCN working/collaboration arrangements in England	RQ2
	Contextual information	Background and wider contextual information with regard to landscape of where the vertical integration model has been implemented	RQ2
	Current model & maturity	Please describe the current model of the acute trust/health board taking over and directly managing general practice services. When did it first take place?	RQ2

Integrative themes	Sub-codes	Description of sub-codes	Research question
	Current stakeholders	Who are the current stakeholders involved? Are these different to those involved in the implementation?	RQ2
	Number of practices	How many practices are involved? Has this increased/decreased since implementation?	RQ2
	Selection process	How are practices selected to join the model? Against what criteria are they measured?	RQ2
Transitioning: from GMS to sub-contracted provider of primary care			RQ3
	Contracts	Who's holding the AMS/GMS contract?	RQ3
	GP property	Who now owns/leases general practice properties?	RQ3
	Impact amongst GPs	How has the vertical integration model impacted governance, contractual and legal arrangements amongst general practices?	RQ3

Integrative themes	Sub-codes	Description of sub-codes	Research question
	Impact between acute trust or health board & GPs	How has the vertical integration model impacted governance, contractual and legal arrangements between acute trust/health board and the general practices?	RQ3
	Indemnity	What changes have occurred (if any) with regard to indemnity?	RQ3
	Limited company	Was there creation of a limited company?	RQ3
	Regulation	New governance and management teams providing oversight to integration. The nature of governance and oversight provided by the vertical integration model e.g. Care Quality Commission	RQ3
	Risk management	What about risk management both on behalf of the acute trust/health board and general practices themselves?	RQ3
	Culture	The lack of understanding of the working of primary care compared to secondary care.	RQ3

Integrative themes	Sub-codes	Description of sub-codes	Research question
	Back office	Is there sharing of 'back office' functions?	RQ3
	Implementing vertical integration	Could you please describe the process of implementing vertical integration in your area? How has the process evolved?	RQ3
	New management board	Has there been an introduction of a new board over-seeing management/performance of integration? Who is involved? Frequency of meetings?	RQ3
	Tensions	Have there been issues of tension amongst stakeholders?	RQ3
Impact on patient management: Changes to primary and secondary care delivery			RQ4 and RQ5
	Differential impact	Does the impact differ for patients from vertical integration practices compared to those registered with non-vertical integration practices? Are patients	RQ4 and RQ5

Integrative themes	Sub-codes	Description of sub-codes	Research question
		experiencing something new/different?	
	Health service provision	How has the delivery of services changed in given settings (primary and secondary care, and wider community services)?	RQ4 and RQ5
	Pathways & processes	What changes have there been with regard to referral pathways/ processes? (If not now, will it change in the future?)	RQ4 and RQ5
	Patient access	What has been the impact on patients accessing services across the care interface?	RQ4 and RQ5
	Patient management	Has integration led to changes to how certain groups of patients are managed?	RQ4 and RQ5
	Career progression	Has the introduction of vertical integration impacted upon career progression (e.g. early career GPs)?	RQ4 and RQ5
	Challenges	What have been some of the key changes which staff have noticed in their	RQ4 and RQ5

Integrative themes	Sub-codes	Description of sub-codes	Research question
		everyday working practices?	
	Impact on recruitment & retention	What has been the impact of the recruitment and retention across both primary and secondary care?	RQ4 and RQ5
	Job satisfaction	Has working within a vertical integration model had any impact upon job satisfaction amongst staff?	RQ4 and RQ5
	New personnel	Has there been an introduction of new personnel? - new types of staff/roles - additional staff numbers	RQ4 and RQ5
Measuring success and identifying the unintended impact of integration			RQ6
	Leavers & joiners	Why have some practices decided to leave or join?	RQ6
	Drawbacks	Are there any financial drawbacks of this model?	RQ6
	Financial implications	Financial implications of this model?	RQ6

Integrative themes	Sub-codes	Description of sub-codes	Research question
	General practices	What has been the financial performance of general practices?	RQ6
	Incentives	What are the financial incentives?	RQ6
	One-off costs	What have been the one-off cost implications for trusts/boards?	RQ6
	Ongoing costs	What have been the ongoing cost implications for trusts/boards?	RQ6
	Next steps	How are you planning to further develop the vertical integration model (what are the next steps)?	RQ6
	Intended outcomes	What are the intended outcomes (clinical and non-clinical)?	RQ6
	Short & long term	How is progress and impact being tracked (using what data) for the short and long term?	RQ6
	Success	How is ‘success’ being determined and/or measured? What process measures are being considered?	RQ6

Integrative themes	Sub-codes	Description of sub-codes	Research question
	Unintended outcomes	Have any unintended outcomes occurred? What are they?	RQ6
	Advice	What advice would you give such prospective areas if they were thinking about adopting this model of vertical integration?	RQ6
	Express of interest	Have any other trusts/health boards expressed interest in adopting a similar vertical integration model?	RQ6
Miscellaneous			
	Further comments	Is there anything else you wanted to tell us about that has not already been covered in the interview?	
	Gold dust	Great quote to use in the final report	
	Site, participant & date	Which site is the interview relevant to? Who is being interviewed? Date of interview?	
	Participant role & stakeholder type	What is your job title? Please describe your current role and key responsibilities? How long	

Integrative themes	Sub-codes	Description of sub-codes	Research question
		have you been in your role? Stakeholder category?	
